# Phylodynamics of HIV-1 Circulating Recombinant Forms 12_BF and 38_BF in Argentina and Uruguay

**DOI:** 10.1186/1742-4690-7-22

**Published:** 2010-03-22

**Authors:** Gonzalo Bello, Paula C Aulicino, Dora Ruchansky, Monick L Guimarães, Cecilio Lopez-Galindez, Concha Casado, Hector Chiparelli, Carlos Rocco, Andrea Mangano, Luisa Sen, Mariza G Morgado

**Affiliations:** 1 Laboratório de AIDS & Imunologia Molecular, Instituto Oswaldo Cruz - FIOCRUZ, Rio de Janeiro, Brazil; 2 Laboratorio de Biología Celular y Retrovirus-CONICET, Hospital de Pediatría J P Garrahan, Buenos Aires, Argentina; 3 Laboratorio Nacional de Referencia VIH-SIDA, Servicio Nacional de Laboratorios de Salud Publica - MSP, Montevideo, Uruguay; 4 Servicio de Virología Molecular, Centro Nacional de Microbiología, Instituto de Salud Carlos III, Madrid, Spain

## Abstract

**Background:**

Although HIV-1 CRF12_BF and CRF38_BF are two epidemiologically important recombinant lineages circulating in Argentina and Uruguay, little is known about their population dynamics.

**Methods:**

A total of 120 "CRF12_BF-like" and 20 "CRF38_BF-like" *pol *recombinant sequences collected in Argentina and Uruguay from 1997 to 2009 were subjected to phylogenetic and Bayesian coalescent-based analyses to estimate evolutionary and demographic parameters.

**Results:**

Phylogenetic analyses revealed that CRF12_BF viruses from Argentina and Uruguay constitute a single epidemic with multiple genetic exchanges among countries; whereas circulation of the CRF38_BF seems to be confined to Uruguay. The mean estimated substitution rate of CRF12_BF at *pol *gene (2.5 × 10-3 substitutions/site/year) was similar to that previously described for subtype B. According to our estimates, CRF12_BF and CRF38_BF originated at 1983 (1978-1988) and 1986 (1981-1990), respectively. After their emergence, the CRF12_BF and CRF38_BF epidemics seem to have experienced a period of rapid expansion with initial growth rates of around 1.2 year^-1 ^and 0.9 year^-1^, respectively. Later, the rate of spread of these CRFs_BF seems to have slowed down since the mid-1990s.

**Conclusions:**

Our results suggest that CRF12_BF and CRF38_BF viruses were generated during the 1980s, shortly after the estimated introduction of subtype F1 in South America (~1975-1980). After an initial phase of fast exponential expansion, the rate of spread of both CRFs_BF epidemics seems to have slowed down, thereby following a demographic pattern that resembles those previously reported for the HIV-1 epidemics in Brazil, USA, and Western Europe.

## Background

The AIDS epidemic in South America is caused by multiple HIV-1 group M subtypes including subtypes B, F1, and C, in addition to BF1 and BC recombinant forms. The BF1 recombinants represent the most widespread genetic form after subtype B and reach a high prevalence (10%-50%) in countries from the Southern Cone (Argentina, Brazil, Chile, Paraguay, and Uruguay) [1-14].

Genetic characterization of BF1 recombinants in South America revealed some important differences across countries. Although four distinct BF1 circulating recombinant forms (CRFs) have been described in Brazil to date (CRF28_BF, CRF29_BF, CRF39_BF, and CRF40_BF) [15,16], the Brazilian BF1 epidemic is largely dominated by a variety of unique recombinants forms (URFs) that do not share a common recombinant ancestor [7,10,17-19]. In contrast, the Argentine BF1 epidemic comprises the widespread CRF12_BF and several URFs with a CRF12-related structure [6,8,20,21]. The molecular epidemiology of HIV-1 in Uruguay is not so well characterized, but two previous studies suggested that BF1 recombinants circulating in this country are similar to those described in Argentina [3,20]. Very recently, a novel CRF38_BF1 was described among Uruguayan HIV-1 isolates, indicating that other BF1 recombinants besides CRF12_BF have gained epidemic importance in this country [14].

Previous studies performed by our group suggest that the HIV-1 subtype F1 and BF1 epidemics in South America were initiated after the introduction of a single F1 strain into Brazil between the middle and late 1970s [22-24]. After its introduction, this founder subtype F1 strain probably recombined with the local subtype B virus generating the large diversity of CRFs_BF1 and URFs_BF1 currently observed in the continent [24]. Based on monophyletic clustering and coincident recombination breakpoints, it was suggested that most BF1 recombinants circulating in Argentina and Uruguay derived from a common recombinant ancestor [21,24,25].

To date, however, very little is known about the evolutionary history and epidemic potential of the diverse BF1 recombinants that have expanded in the South American population. Only one previous study was conducted on a small number (*n *= 40) of CRF12_BF-like *vpu *sequences from a vertically infected population in Argentina [26]. This study estimated the age of the most recent common ancestor (MRCA) of those CRF12_BF-like viruses between 1981 and 1996, and further suggests an extremely rapid spread of the CRF12_BF-like recombinant viruses, compatible with the demographic pattern of explosive population growth observed in this pediatric population at the start of the epidemic.

The objective of the present study was to reconstruct the evolutionary and demographic history of the CRF12_BF circulating in Argentina and Uruguay through the analysis of a large data set (*n *= 120) of CRF12_BF-like *pol *sequences recovered from adults and children living in both countries. In addition, we also analyzed a small data set (*n *= 20) of CRF38_BF-like *pol *sequences to estimate the age and demographic history of the CRF38_BF epidemic spreading in Uruguay. This data represent an excellent opportunity to explore potential CRF-specific and regional-specific differences in the patterns of HIV-1 epidemic growth in South America.

## Methods

### Study population

**Figure 1:**
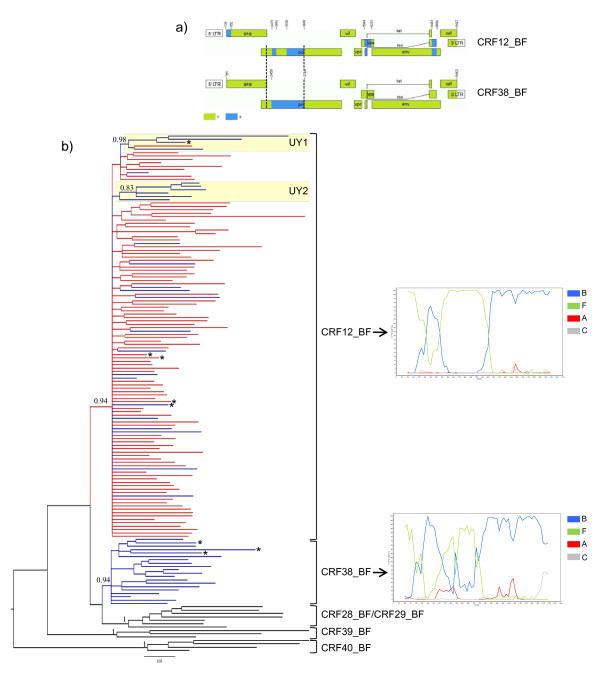
**Virus analyses**. a) Genomic mosaic structure of CRF12_BF and CRF38_BF viruses. Green, subtype F1; blue subtype B; white, unknown subtype. Numbers above breakpoints refer to nucleotide positions in the HXB2 genome. Vertical dotted lines indicate the *pol *gene fragment (nucleotides 2266-3705) used in the present study. b) Majority-rule Bayesian consensus tree of the *pol *gene of HIV-1 CRFs_BF circulating in Argentina (red), Uruguay (blue), and Brazil (black). Posterior probability values are indicated only at key nodes. Brackets indicate the monophyletic clusters formed by each CRF. Boxes indicate the two Uruguayan sub-cluters identified within the CRF12_BF clade. Positions of the full-length characterized CRF12_BF and CRF38_BF reference sequences are marked with asterisks. The tree was rooted on midpoint and horizontal branch lengths are drawn to scale with the bar at the bottom indicating 0.03 nucleotide substitutions per site. Representative bootscanning plots of the *pol *gene fragment of CRF12_BF (A32879) and CRF38_BF (UY03_3389) reference sequences are depicted on the right. Reference sequences used for these analyses were as follows: subtype B (BZ126, blue), subtype F1 (BZ167, green), subtype C (92BR025, gray) and subtype A1 (U455, red).

A total of 66 and 17 samples with a CRF12_BF-like and CRF38_BF-like mosaic pattern at the *pol *gene, respectively, were selected from HIV-1-infected patients residing in Argentina and Uruguay who had previously been analyzed in two independent studies. The first study analyzed the genetic structure of BF1 *pol *recombinant sequences collected between 1997 and 2008 from HIV-1 infected children followed up at the "Hospital de Pediatria Garrahan" in Buenos Aires, Argentina, identifying 43 samples with a CRF12_BF-like mosaic pattern (Aulicino *et al*, publication in progress). The second study assessed the genetic diversity in a group of BF1 *pol *recombinant samples collected between 1997 and 2009 from HIV-1 infected adults and children residing in Uruguay, identifying 23 samples with a CRF12_BF-like mosaic structure and 17 samples with a CRF38_BF-like mosaic pattern (Ruchansky *et al*, publication in progress). These unpublished sequences were combined with CRF12_BF (Argentina *n *= 3; Uruguay *n *= 2) and CRF38_BF (Uruguay *n *= 3) reference sequences, and CRF12_BF-like *pol *sequences (Argentina *n *= 48; Uruguay *n *= 1) from adults patients with known sampling dates retrieved from the Los Alamos HIV Sequence Database http://www.hiv.lanl.gov/content/index, as described in Table 1. Sequences were excluded if they originated from the same patient or from individuals known to be related by direct transmission. The sequences were ~1440 bp long and covered the protease (*PR*) and part of the reverse transcriptase (*RT*) genes (nucleotides 2266-3705 relative to the HXB2 clone), encompassing the recombinant fragments of the CRF12_BF and CRF38_BF at *pol *gene (Fig. 1a). Nucleotide sequences were aligned using CLUSTAL X program [27]. All positions with alignment gaps were excluded from analyses.

**Table 1:** HIV-1 CRF12_BF and CRF38_BF data sets.

CRF_BF	Year	New	Database	Total	References
**12**	1997	7	3	10	[21]
	1998	9	-	9	
	1999	4	8	12	[20,21]
	2000	5	-	5	
	2001	0	20	20	[6]
	2002	6	-	6	
	2003	8	12	20	[9]
	2004	6	11	17	[8]
	2005	5	-	5	
	2006	4	-	4	
	2007	2	-	2	
	2008	10	-	10	
	**Total**	**66**	**54**	**120**	

**38**	1997	1	-	1	
	1998	2	-	2	
	1999	2	-	2	
	2000	1	-	1	
	2003	8	1	9	[14]
	2004	1	1	2	[14]
	2005	1	1	2	[14]
	2009	1	-	1	
	**Total**	**17**	**3**	**20**	

### Characterization of "CRF-like" recombinant profiles

Two strategies were used to characterize the HIV-1 *pol *sequences used in the present study as CRF12_BF-like or CRF38_BF-like recombinants:

1) First, the recombination breakpoints of each sequence were identified by Bootscanning using Simplot version 3.5.1 [28]. Bootstrap values supporting branching with reference sequences were determined in Neighbor-Joining (NJ) trees constructed using the K2-P [29] nucleotide substitution model, based on 100 re-samplings, with a 200 bp sliding window moving in steps of 20 bases. Individual query sequences were compared to representative reference sequences of HIV-1 subtypes A1, B, C, and F1. Sequences were considered to have a "CRF-like" profile if recombination sites exactly match those identified in CRF12_BF and CRF38_BF reference sequences.

2) Second, Bayesian and Maximum Likelihood (ML) phylogenetic trees for the final *pol *alignment including all CRF-like sequences were built to confirm the overall topology and strong support of each CRF clade. Phylogenetic trees were constructed under the GTR [30] nucleotide substitution model, with a gamma-distribution model of among site rate heterogeneity and a proportion of invariable sites (GTR+I+Γ) selected using the Modeltest program [31]. A Bayesian phylogeny was estimated using MrBayes [32]. Two runs of four chains each were run for 50 × 10^6 ^generations, with a burn-in of 5 × 10^6 ^generations. Convergence of parameters was assessed by calculating the Effective Sample Size (ESS) using TRACER v1.4 [33], after excluding an initial 10% for each run. All parameter estimates for each run showed ESS values >100. ML trees were reconstructed with PhyML [34] using an online web server [35]. Heuristic tree searches were performed using the SPR branch-swapping algorithm, and the approximate likelihood-ratio test (aLRT) based on a Shimodaira-Hasegawa-like procedure was used as a statistical test to calculate branch support. Trees were visualized with the FigTree v1.1.2 program (available at http://tree.bio.ed.ac.uk/software/figtree/).

### Estimation of evolutionary rates, dates, and demographic history

The evolutionary rate (*μ*, units are nucleotide substitutions per site per year), the age of the most recent common ancestor (*T*_mrca_, years), and the mode and rate (*r*, years^-1^) of population growth for the CRF12_BF and CRF38_BF strains were estimated using BEAST v1.4.7 [36,37]. Evolutionary and demographic parameters of CRF12_BF were estimated under a chronological time-scale employing the dates of sample collection. The low number of CRF38_BF sequences analyzed (i.e., 20 sequences), however, was not sufficient to obtain an accurate estimate of the evolutionary rate of this lineage. Therefore, the rates of evolution at *pol *(*PR/RT*) gene previously estimated for other HIV-1 group M subtypes (1.5 × 10^-3 ^- 2.5 × 10^-3 ^substitutions/site/year) [38-41] were incorporated as a prior probability distribution in the analysis of this CRF. Estimations of evolutionary and demographic parameters involved two steps. First, the Bayesian skyline plot method [42] was used to estimate *μ*, the *T*_mrca_, and the change in effective population size through time. Second, two different demographic models for each data set were compared: exponential and logistic growth; and estimates of the population growth rate were then obtained under the model that provided the best fit to the demographic signal in each data set. Model comparisons in a Bayesian framework were performed by calculating the Bayes Factor (BF) [43] with TRACER v1.4. Analyses were performed using the GTR+I+Γ nucleotide substitution model under either strict or uncorrelated Lognormal relaxed [44] molecular clock models. Two separate MCMC chains were run for 10-50 × 10^6 ^generations, with a burn-in of 1-5 × 10^6^. BEAST output was analysed using TRACER v1.4, with uncertainty in parameter estimates reflected in the 95% Highest Probability Density (HPD) intervals. Convergence of parameters was assessed through the ESS, with all parameter estimates for each run showing ESS values >100. A graphical representation of the effective number of infections through time was generated by using programs TRACER v1.4 and Prism 4 (GraphPad Software). Posterior trees samples from BEAST runs were summarized using TreeAnnotator v1.4.7 (available from http://beast.bio.ed.ac.uk) to generate time-scaled maximum clade credibility trees.

## Results

A total of 115 *pol *sequences (Argentina = 91, Uruguay = 24) with a "CRF12_BF-like" recombination profile, and 17 *pol *sequences (Uruguay) with a "CRF38_BF-like" recombinant pattern were identified by Bootscanning analyses. These sequences were aligned with reference sequences of CRF12_BF, CRF38_BF and Brazilian CRFs_BF1, and analyzed using Bayesian and ML approaches. Both phylogenetic approaches showed that the CRF12_BF-like and CRF38_BF-like *pol *sequences segregated with their respective CRF reference sequences in two well supported monophyletic groups characterized by unique recombination profiles, confirming the common ancestry of each CRF (Fig. 1). Of note, Simplot analysis suggests that the CRF38_BF presents a more complex BF1 mosaic pattern at the *PR/RT *genomic region than that previously described [14], characterized by the presence of small subtype F1 fragments between positions 2640 and 3020 relative to HXB2 (Fig. 1). More detailed analysis of the *pol *genomic region should be performed in order to determine the precise mosaic structure of the CRF38_BF at that region.

Within the CRF12_BF clade, two strongly supported Uruguayan subclusters comprising four (cluster UY-1) and six (cluster UY-2) viruses were identified in both Bayesian (posterior probability [PP] > 0.80) (Fig. 1) and ML (aLTR > 0.70) phylogenetic trees (data not shown). Most (61%) CRF12_BF Uruguayan sequences, however, were randomly interspersed among Argentine sequences, which provides evidence against the existence of a specific Argentine or Uruguayan CRF12_BF lineage. This contrasts with the circulation of CRF38_BF which seems to be restricted to Uruguay, as no strains with a CRF38_BF-like structure were identified after analysis of more than 300 BF1 recombinant *pol *sequences from Argentina (74 unpublished sequences and 249 sequences retrieved from the Los Alamos HIV Sequence Database).

Bayesian MCMC analyses under a skyline tree prior were used to estimate the time-scale of the CRF12_BF and CRF38-BF epidemics. The mean estimated evolutionary rate for the CRF12_BF *pol *gene was 2.4 × 10^-3 ^subst./site/year, under both strict and relaxed molecular clock models (Table 2). The median rate of evolution for the CRF38_BF *pol *gene was 1.8 × 10^-3 ^subst./site/year (strict clock) and 1.9 × 10^-3 ^(relaxed clock), although the 95% HPD intervals of those estimates almost coincide with the informative prior interval (Table 2), indicating that not much information was added by the data. Considering these substitution rates, the median *T*_mrca _of the CRFs was estimated at 1982 (strict clock) and 1983 (relaxed clock) for the CRF12_BF, and 1985 (strict clock) and 1986 (relaxed clock) for the CRF38_BF (Table 2).

**Table 2:** Bayesian estimates of evolutionary parameters of the HIV-1 CRF12_BF and CRF38_BF epidemics.

Subtype	Gene	Coalescent	Molecular clock	*μ*	*T*mrca
CRF12_BF	*pol*	Bayesian Skyline	Strict	2.4 × 10^-3^ (1.9 × 10^-3^-2.9 × 10^-3^)	1982 (1976-1986)
			
			Relaxed	2.4 × 10^-3^ (1.8 × 10^-3^-3.1 × 10^-3^)	1983 (1978-1988)
		
		Logistic growth	Strict	2.4 × 10^-3^ (1.9 × 10^-3^-2.8 × 10^-3^)	1982 (1978-1986)
			
			Relaxed	2.5 × 10^-3^ (1.9 × 10^-3^-3.0 × 10^-3^)	1983 (1979-1987)

CRF38_BF ^*a*^	*pol*	Bayesian Skyline	Strict	1.8 × 10^-3^ (1.5 × 10^-3^-2.2 × 10^-3^)	1985 (1977-1989)
			
			Relaxed	1.9 × 10^-3^ (1.5 × 10^-3^-2.3 × 10^-3^)	1986 (1981-1990)
		
		Logistic growth	Strict	1.8 × 10^-3^ (1.5 × 10^-3^-2.1 × 10^-3^)	1985 (1980-1989)
			
			Relaxed	1.8 × 10^-3^ (1.4 × 10^-3^-2.3 × 10^-3^)	1986 (1981-1990)

Estimates of the mean evolutionary rate (*μ*, substitutions site^-1 ^year^-1^) and median time of the most recent common ancestor (*T*mrca, year) of the HIV-1 CRF12_BF and CRF38_BF epidemics (95% HPD intervals in parentheses). The results reported are the combined estimates of two independent runs. ^a ^Informative prior distribution of *μ *(1.5 × 10^-3^-2.5 × 10^-3^) for the CRF38_BF *pol *data set was selected from: Hué *et al*. [38], Salemi *et al*.[39], Bello *et al*.[40], and Passaes *et al*. [41].

**Figure 2:**
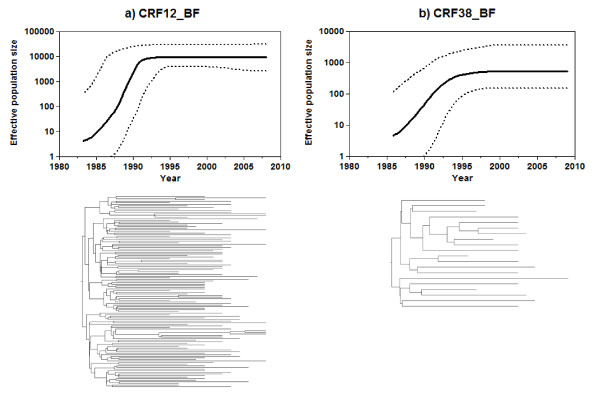
**Time-scaled Bayesian phylogenies and corresponding Bayesian skyline plots (BSP) for the HIV-1 CRF12_BF (a) and CRF38_BF (b) epidemics**. Time-scaled phylogenies and BSP were generated under a relaxed molecular clock model using BEAST. Branch lengths of the trees represent time (see the time scale at the X-axis of each graph). BSP represent estimates of effective number of infections (Y-axis; log_10 _scale) through time (X-axis; calendar years). Median (solid line) and upper and lower 95% HPD (dashed lines) estimates of the effective number of infections are shown in each graph.

Bayesian skyline plot analyses were also used to infer the demographic history of South American CRF_BF epidemics. According to this analysis the CRF12_BF epidemic experienced a fast exponential growth during the first 10-15 years followed by a more recent decline in growth rate since the mid-1990s (Fig. 2a). A very similar demographic pattern was observed for the CRF38_BF, showing that after an initial period of exponential growth of ~10 years the growth rate of this CRF epidemic also slowed around the mid-1990s (Fig. 2b). These results suggest that a model of logistic population growth fits the demographic information contained in the CRF12_BF and CRF38_BF data sets better than the exponential growth model.

To test this hypothesis, approximate marginal log likelihoods for the logistic and exponential growth models were calculated. The analysis of BF clearly showed that, for both CRFs, the model of logistic population growth was strongly supported over the exponential growth model, under either a strict or a relaxed molecular clock (Table 3). On the other hand, models assuming a relaxed molecular clock fit the CRF12_BF and CRF38_BF data sets better than models enforcing a strict molecular clock (Table 3) indicating that substitution rate varies among branches consistent with other HIV-1 studies [39,45-47]. Indeed, the coefficients of variation under the relaxed clock model were higher than zero for both CRF12_BF (*mean *= 0.21, 95% HPD: 0.16-0.26) and CRF38_BF (*mean *= 0.28, 95% HPD: 0.10-0.46).

**Table 3:** Bayes Factors (BF) between exponential (Exp) and logistic (Log) growth demographic models for the HIV-1 CRF12_BF and CRF38_BF *pol *data sets.

Subtype	Model comparison	ln (BF)^a^	Evidence against H_0_^b^
CRF12_BF	Exp (H_0_) vs Log (H_1_) Strict clock	111.7 (0.6)	Decisive
	
	Exp (H_0_) vs Log (H_1_) Relaxed clock	142.0 (0.7)	Decisive
	
	Log Strict (H_0_) vs Relaxed (H_1_) clock	48.0 (0.5)	Decisive

CRF38_BF	Exp (H_0_) vs Log (H_1_) Strict clock	6.9 (0.3)	Decisive
	
	Exp (H_0_) vs Log (H_1_) Relaxed clock	10.5 (0.4)	Decisive
	
	Log Strict (H_0_) vs Relaxed (H_1_) clock	8.7 (0.3)	Decisive

^*a *^ln (BF) = Bayes Factor is the difference (in ln space) of the marginal likelihood of null (H_0_) and alternative (H_1_) model. The SE of the estimates is given in parenthesis and was estimated using 1000 bootstrap replicates. ^*b *^Evidence against H_0 _was assessed in the following way: ln (BF) < 0 indicates no evidence against the null model; ln (BF) between 0-2.3 indicates weak evidence against the null model, ln (BF) between 2.3-3.4 indicates strong evidence against the null model; ln (BF) between 3.4-4.6 indicates very strong evidence against the null model; and ln (BF) > 4.6 indicates decisive evidence against the null model.

A coalescent model of logistic growth was then used to estimate the initial growth rate of South American CRF_BF epidemics. Evolutionary parameters estimated under the logistic growth tree prior were almost identical to those estimated with Bayesian skyline (Table 2). The mean estimated growth rate of CRF12_BF epidemic was 1.08 year^-1 ^(strict clock), and 1.22 year^-1 ^(relaxed clock). This rate corresponds to a mean epidemic doubling time of around six months (Table 4). The mean growth rate of the CRF38_BF epidemic was estimated at 0.83 year^-1 ^(strict clock) and 0.92 year^-1 ^(relaxed clock), which corresponds to a mean epidemic doubling time of <1 year (Table 4).

**Table 4:** Bayesian estimates of population dynamic parameters of the HIV-1 CRF12_BF and CRF38_BF epidemics.

Subtype	Demographic model	Molecular Clock	Gene	*r*	λ
CRF12_BF	Logistic growth	Strict	*pol (PR-RT)*	1.08 (0.79-1.44)	0.64 (0.48-0.88)
				
		Relaxed		1.22 (0.85-1.64)	0.57 (0.42-0.81)

CRF38_BF	Logistic growth	Strict	*pol (PR-RT)*	0.83 (0.31-1.81)	0.83 (0.38-2.24)
				
		Relaxed		0.92 (0.41-1.75)	0.75 (0.40-1.69)

CRF12_BF^*a*^	Logistic growth	Relaxed	*vpu*	2.24 (0.21-4.56)	0.31 (0.15-3.30)

B^*b*^	Logistic growth		*env (C2-V3)*	0.46 (0.33-0.59)	1.51 (1.22-2.10)
		Strict			
			*pol (PR-RT)*	0.56 (0.35-0.80)	1.24 (0.87-1.98)

F^*b*^	Logistic growth		*env (C2-V3)*	0.61 (0.40-0.86)	1.14 (0.81-1.73)
		Strict			
			*pol (PR-RT)*	0.59 (0.31-0.92)	1.17 (0.75-2.24)

C^*c*^	Logistic growth	Strict	*pol (RT)*	0.70 (0.41-1.00)	0.99 (0.69-1.69)
				
		Relaxed		0.81 (0.40-1.26)	0.86 (0.55-1.73)

CRF31_BC^*c*^	Logistic growth	Strict	*pol (RT)*	1.26 (0.61-2.10)	0.55 (0.33-1.14)
				
		Relaxed		1.27 (0.44-2.26)	0.55 (0.31-1.57)

Estimates of the median growth rate (*r*, yr^-1^) and epidemic doubling time (λ, yr) for the HIV-1 CRF12_BF and CRF38_BF epidemics (95% HPD in parentheses). Growth rate estimates were used to calculate the time taken for the epidemic to double in size (λ) using the relation λ = ln(2)/r. ^a ^Data from Aulicino *et al*. [26]. ^b^Data from Bello *et al*. [23]. ^c ^Data from Bello *et al*. [47].

## Discussion

We have performed an extensive study of the evolutionary history and population dynamics of the two most prevalent HIV-1 BF1 recombinant strains (CRF12_BF and CRF38_BF) spreading in South America. The CRF12_BF was the first CRF to be described in South America among samples isolated from Argentina and Uruguay [20], and is responsible for a significant part of the HIV-epidemics in those countries. Our phylogenetic analyses of recombinant *pol *sequences with a CRF12-like structure showed for the first time that CRF12_BF viruses spreading in Argentina and Uruguay constitute a single epidemic with evidences of multiple genetic exchanges among countries. In contrast, circulation of the recently described CRF38_BF [14] seems to be restricted to Uruguay as no strains with a CRF38_BF-like *pol *mosaic pattern were observed after screening of a large number (*n *> 300) of BF1 recombinant sequences from Argentina.

According to our estimates, the mean *T*_mrca _of CRF12_BF and CRF38_BF was 1982-1983 and 1985-1986, respectively; only a few years later than the mean estimated onset date of subtype F1 spread in Brazil (1976-1978) [23,24]. These mean estimates are fully consistent with epidemiological data that revealed that BF1 recombinants related to the CRF12_BF have been in circulation in Argentine children since the mid 1980s [48]. In agreement with this study, we identified five Argentine children born between 1987 and 1989 infected by CRF12-like BF viruses, and one Uruguayan child born in 1987 infected by a CRF38-like BF strain.

Our study showed that the substitution rate of CRF12_BF viruses at *pol *gene (2.5 × 10^-3 ^subst./site/year) is similar to rates previously described for HIV-1 subtypes B, C, and CRF31_BC [38,40,41], pointing to no major differences in evolution rate among HIV-1 strains circulating in South America. By contrast, some differences in epidemic growth rate may have existed across distinct HIV-1 variants circulating in South America. The estimated initial growth rate of the CRF12_BF epidemic in Argentina and Uruguay was higher than that described for Brazilian subtype B, C, and F1 epidemics [23,47], and similar to that recently described for the CRF31_BC viruses circulating in southern Brazil [47] (Table 4); supporting the notion of a fast initial spread of CRF12_BF-like viruses [26]. The initial expansion rate of CRF38_BF also seems to be higher than those described for subtypes B and F1, although the 95% HPD interval of such an estimate was quite large (Table 4).

The rapid initial growth rate of CRF12_BF, CRF31_BC, and CRF38_BF epidemics in South America may indicate that these CRFs displayed a higher fitness and/or transmissibility than parental HIV-1 subtypes. Alternatively, variations in the initial growth rate of HIV-1 variants may reflect differences in the susceptible populations that characterized the initial spread of each strain. Of note, the BF recombinants have been associated with injection drug use populations in Argentina and Uruguay [3,5], and introduction of a BF1 recombinant virus in such highly connected networks may explain the emergence and rapid initial dissemination of the CRF_BF1 viruses.

After the initial period of fast exponential growth, the expansion rate of the CRF12_BF and CRF38_BF epidemics slowed down since the mid 1990s. The same demographic pattern was described for HIV-1 epidemics in Brazil, USA, and some European countries [23,38,47,49-51]. Such a recent decline in the growth rate of these HIV-1 epidemics may be the consequence of adequate prevention campaigns implemented after the official report of the first AIDS cases in the early 1980s, and/or the result of a saturation of high-risk transmission networks that are loosely connected with low-risk subgroups that exhibit modest levels of HIV-1 infection [52].

The overall demographic pattern of the CRF12_BF epidemic in Argentina and Uruguay observed in this study is similar with that previously described for the CRF12_BF epidemic in Argentine children [26]; and our mean estimates of *T*_mrca _and population growth rate fell within the 95% HPD interval of the previous estimates (Table 4). The confidence intervals of these new estimates, however, were much narrower than those previously obtained (Table 4), indicating that the use of a larger data set have substantially improved the accuracy of parameter estimation. In agreement with this idea, the small CRF38 data set was associated with much larger confidence intervals of demographic parameter estimates than those obtained for the larger CRF12_BF data set. Thus, more precise estimations of demographic parameters of CRF38_BF will require the analysis of a larger number of sequences.

## Conclusions

Our results suggest that CRF12_BF and CRF38_BF viruses were rapidly generated after the introduction of subtype F1 into South America and have been circulating in the continent over the last 25 years. Both CRFs seem to have spread exponentially at a fast rate during the 1980s and the early 1990s; but the rate of spread of the CRF12_BF and CRF38_BF viruses slowed down since the mid 1990s. Despite similar emergence dates and demographic histories, CRF12_BF have been widely disseminated in Argentina and Uruguay, whereas the CRF38_BF circulation was found limited to Uruguay. Determination of the factors that have shaped the pattern and rate of spread of distinct HIV-1 variants in South America is of paramount importance to understand the epidemic potential of these variants.

## Competing interests

The authors declare that they have no competing interests.

## Authors' contributions

GB, PCA, DR, MLG, and MM conceived and designed the study. DR and HC were responsible for patients' recruitment and sample collection in Uruguay. PCA, CR, AM, and LS were responsible for patients' recruitment and sample collection in Argentina. DR, PCA, CLG, CC, MLG and GB performed the characterization of samples in the *pol *region. GB performed the phylogenetic and coalescent analyses. GB wrote the first draft and all authors contributed to the final version of the paper.

## References

[B1] Thomson MM Villahermosa ML Vazquez-de-Parga E Cuevas MT Delgado E Manjon N Medrano L Perez-Alvarez L Contreras G Carrillo MG Salomón H Nájera R Widespread circulation of a B/F intersubtype recombinant form among HIV-1-infected individuals in Buenos Aires, Argentina AIDS 2000 14 897 899 10.1097/00002030-200005050-00020 10839601

[B2] Guimaraes ML dos Santos Moreira A Loureiro R Galvao-Castro B Morgado MG High frequency of recombinant genomes in HIV type 1 samples from Brazilian southeastern and southern regions AIDS Res Hum Retroviruses 2002 18 1261 1269 10.1089/088922202320886307 12487814

[B3] Hierholzer J Montano S Hoelscher M Negrete M Hierholzer M Avila MM Carrillo MG Russi JC Vinoles J Alava A Acosta ME Gianella A Andrade R Sanchez JL Carrion G Sanchez JL Russell K Robb M Birx D McCutchan F Carr JK Molecular Epidemiology of HIV Type 1 in Ecuador, Peru, Bolivia, Uruguay, and Argentina AIDS Res Hum Retroviruses 2002 18 1339 1350 10.1089/088922202320935410 12487805

[B4] Teixeira SL Bastos FI Telles PR Hacker MA Brigido LF de FOCA Bongertz V Morgado MG HIV-1 infection among injection and ex-injection drug users from Rio de Janeiro, Brazil: prevalence, estimated incidence and genetic diversity J Clin Virol 2004 31 221 226 10.1016/j.jcv.2004.03.016 15465416

[B5] Espinosa A Vignoles M Carrillo MG Sheppard H Donovan R Peralta LM Rossi D Radulich G Salomon H Weissenbacher M Intersubtype BF recombinants of HIV-1 in a population of injecting drug users in Argentina J Acquir Immune Defic Syndr 2004 36 630 636 10.1097/00126334-200405010-00012 15097307

[B6] Quarleri JF Rubio A Carobene M Turk G Vignoles M Harrigan RP Montaner JS Salomon H Gomez-Carrillo M HIV type 1 BF recombinant strains exhibit different pol gene mosaic patterns: descriptive analysis from 284 patients under treatment failure AIDS Res Hum Retroviruses 2004 20 1100 1107 10.1089/aid.2004.20.1100 15585101

[B7] Brigido LF Franco HM Custodio RM Oliveira CA JL PF Eira M Bergel F Araujo F Carvalheiro JR Rodrigues R Molecular characteristics of HIV type 1 circulating in Sao Paulo, Brazil AIDS Res Hum Retroviruses 2005 21 673 682 10.1089/aid.2005.21.673 16060840

[B8] Gomez-Carrillo M Pampuro S Duran A Losso M Harris DR Read JS Duarte G De Souza R Soto-Ramirez L Salomon H Analysis of HIV type 1 diversity in pregnant women from four Latin American and Caribbean countries AIDS Res Hum Retroviruses 2006 22 1186 1191 10.1089/aid.2006.22.1186 17147509

[B9] Dilernia DA Gomez AM Lourtau L Marone R Losso MH Salomon H Gomez-Carrillo M HIV type 1 genetic diversity surveillance among newly diagnosed individuals from 2003 to 2005 in Buenos Aires, Argentina AIDS Res Hum Retroviruses 2007 23 1201 1207 10.1089/aid.2007.0068 17961105

[B10] Brennan CA Brites C Bodelle P Golden A Hackett J Jr Holzmayer V Swanson P Vallari A Yamaguchi J Devare S Pedroso C Ramos A Badaro R HIV-1 strains identified in Brazilian blood donors: significant prevalence of B/F1 recombinants AIDS Res Hum Retroviruses 2007 23 1434 1441 10.1089/aid.2007.0121 18184087

[B11] Rios M Delgado E Perez-Alvarez L Fernandez J Galvez P de Parga EV Yung V Thomson MM Najera R Antiretroviral drug resistance and phylogenetic diversity of HIV-1 in Chile J Med Virol 2007 79 647 656 10.1002/jmv.20881 17457921

[B12] de Sa-Filho DJ Soares Mda S Candido V Gagliani LH Cavaliere E Diaz RS Caseiro MM HIV type 1 pol gene diversity and antiretroviral drug resistance mutations in Santos, Brazil AIDS Res Hum Retroviruses 2008 24 347 353 10.1089/aid.2007.0203 18327988

[B13] Aguayo N Laguna-Torres VA Villafane M Barboza A Sosa L Chauca G Carrion G Coenca B Perez J Galeano A Bautista CT Sanchez JL Carr JK Kochel T Epidemiological and molecular characteristics of HIV-1 infection among female commercial sex workers, men who have sex with men and people living with AIDS in Paraguay Rev Soc Bras Med Trop 2008 41 225 231 10.1590/S0037-86822008000300001 18719799

[B14] Ruchansky D Casado C Russi JC Arbiza JR Lopez-Galindez C Identification of a new HIV Type 1 circulating recombinant form (CRF38_BF1) in Uruguay AIDS Res Hum Retroviruses 2009 25 351 356 10.1089/aid.2008.0248 19327055

[B15] De Sa Filho DJ Sucupira MC Casiero MM Sabino EC Diaz RS Janini LM Identification of two HIV type 1 circulating recombinant forms in Brazil AIDS Res Hum Retroviruses 2006 22 1 13 10.1089/aid.2006.22.1 16438639

[B16] Guimaraes ML Eyer-Silva WA Couto-Fernandez JC Morgado MG Identification of two new CRF_BF in Rio de Janeiro State, Brazil AIDS 2008 22 433 435 10.1097/QAD.0b013e3282f47ad0 18195572

[B17] Thomson MM Sierra M Tanuri A May S Casado G Manjon N Najera R Analysis of near full-length genome sequences of HIV type 1 BF intersubtype recombinant viruses from Brazil reveals their independent origins and their lack of relationship to CRF12_BF AIDS Res Hum Retroviruses 2004 20 1126 1133 10.1089/aid.2004.20.1126 15585105

[B18] Sa Filho DJ Sanabani S Diaz RS Munerato P Brunstein A Fusuma E Sabino EC Janini LM Analysis of full-length human immunodeficiency virus type 1 genome reveals a variable spectrum of subtypes B and f recombinants in Sao Paulo, Brazil AIDS Res Hum Retroviruses 2005 21 145 151 10.1089/aid.2005.21.145 15725753

[B19] Sanabani S Neto WK Kalmar EM Diaz RS Janini LM Sabino EC Analysis of the near full length genomes of HIV-1 subtypes B, F and BF recombinant from a cohort of 14 patients in Sao Paulo, Brazil Infect Genet Evol 2006 6 5 368 77 10.1016/j.meegid.2006.01.003 16522378

[B20] Carr JK Avila M Gomez Carrillo M Salomon H Hierholzer J Watanaveeradej V Pando MA Negrete M Russell KL Sanchez J Birx DL Andrade R Vinoles J McCutchan FE Diverse BF recombinants have spread widely since the introduction of HIV-1 into South America AIDS 2001 15 F41 47 10.1097/00002030-200110190-00002 11600844

[B21] Thomson MM Delgado E Herrero I Villahermosa ML Vazquez-de Parga E Cuevas MT Carmona R Medrano L Perez-Alvarez L Cuevas L Najera R Diversity of mosaic structures and common ancestry of human immunodeficiency virus type 1 BF intersubtype recombinant viruses from Argentina revealed by analysis of near full-length genome sequences J Gen Virol 2002 83 107 119 11752707 10.1099/0022-1317-83-1-107

[B22] Bello G Guimaraes ML Morgado MG Evolutionary history of HIV-1 subtype B and F infections in Brazil AIDS 2006 20 763 768 10.1097/01.aids.0000216377.84313.52 16514307

[B23] Bello G Eyer-Silva WA Couto-Fernandez JC Guimaraes ML Chequer-Fernandez SL Teixeira SL Morgado MG Demographic history of HIV-1 subtypes B and F in Brazil Infect Genet Evol 2007 7 263 270 10.1016/j.meegid.2006.11.002 17150415

[B24] Aulicino PC Bello G Rocco C Romero H Mangano A Morgado MG Sen L Description of the First Full-Length HIV Type 1 Subtype F1 Strain in Argentina: Implications for the Origin and Dispersion of This Subtype in South America AIDS Res Hum Retroviruses 2007 23 1176 1182 10.1089/aid.2007.0038 17961101

[B25] Sierra M Thomson MM Rios M Casado G Castro RO Delgado E Echevarria G Munoz M Colomina J Carmona R Vega Y Parga EV Medrano L Pérez-Alvarez L Contreras G Nájera R The analysis of near full-length genome sequences of human immunodeficiency virus type 1 BF intersubtype recombinant viruses from Chile, Venezuela and Spain reveals their relationship to diverse lineages of recombinant viruses related to CRF12_BF Infect Genet Evol 2005 5 209 217 10.1016/j.meegid.2004.07.010 15737911

[B26] Aulicino PC Holmes EC Rocco C Mangano A Sen L Extremely rapid spread of human immunodeficiency virus type 1 BF recombinants in Argentina J Virol 2007 81 427 429 10.1128/JVI.01403-06 17050594 PMC1797267

[B27] Thompson JD Gibson TJ Plewniak F Jeanmougin F Higgins DG The CLUSTAL_X windows interface: flexible strategies for multiple sequence alignment aided by quality analysis tools Nucleic Acids Res 1997 25 4876 4882 10.1093/nar/25.24.4876 9396791 PMC147148

[B28] Ray S Simplot v2.5.0 http://sray.med.som.jhmi.edu/SCRoftware/simplot/

[B29] Kimura M A simple method for estimating evolutionary rates of base substitutions through comparative studies of nucleotide sequences J Mol Evol 1980 16 111 120 10.1007/BF01731581 7463489

[B30] Tavaré S Miura RM Some probabilistic and statistical problems in the analysis of DNA sequences. p Some mathematical questions in biology--DNA sequence analysis Providence (RI): American Mathematical Society 1986 57 86

[B31] Posada D Crandall KA MODELTEST: testing the model of DNA substitution Bioinformatics 1998 14 817 818 10.1093/bioinformatics/14.9.817 9918953

[B32] Ronquist F Huelsenbeck JP MrBayes 3: Bayesian phylogenetic inference under mixed models Bioinformatics 2003 19 1572 1574 10.1093/bioinformatics/btg180 12912839

[B33] Rambaut A Drummond A Tracer v1.4 2007 http://beast.bio.ed.ac.uk/Tracer

[B34] Guindon S Gascuel O A simple, fast, and accurate algorithm to estimate large phylogenies by maximum likelihood Syst Biol 2003 52 696 704 10.1080/10635150390235520 14530136

[B35] Guindon S Lethiec F Duroux P Gascuel O PHYML Online--a web server for fast maximum likelihood-based phylogenetic inference Nucleic Acids Res 2005 33 W557 559 10.1093/nar/gki352 15980534 PMC1160113

[B36] Drummond AJ Nicholls GK Rodrigo AG Solomon W Estimating mutation parameters, population history and genealogy simultaneously from temporally spaced sequence data Genetics 2002 161 1307 1320 12136032 10.1093/genetics/161.3.1307 PMC1462188

[B37] Drummond AJ Rambaut A BEAST: Bayesian evolutionary analysis by sampling trees BMC Evol Biol 2007 7 214 10.1186/1471-2148-7-214 17996036 PMC2247476

[B38] Hue S Pillay D Clewley JP Pybus OG Genetic analysis reveals the complex structure of HIV-1 transmission within defined risk groups Proc Natl Acad Sci USA 2005 102 4425 4429 10.1073/pnas.0407534102 15767575 PMC555492

[B39] Salemi M de Oliveira T Ciccozzi M Rezza G Goodenow MM High-Resolution Molecular Epidemiology and Evolutionary History of HIV-1 Subtypes in Albania PLoS ONE 2008 3 e1390 10.1371/journal.pone.0001390 18167549 PMC2148102

[B40] Bello G Passaes CP Guimaraes ML Lorete RS Matos Almeida SE Medeiros RM Alencastro PR Morgado MG Origin and evolutionary history of HIV-1 subtype C in Brazil AIDS 2008 22 1993 2000 10.1097/QAD.0b013e328315e0aa 18753928

[B41] Passaes CP Bello G Lorete RS Matos Almeida SE Junqueira DM Veloso VG Morgado MG Guimaraes ML Genetic characterization of HIV-1 BC recombinants and evolutionary history of the CRF31_BC in Southern Brazil Infect Genet Evol 2009 9 474 482 10.1016/j.meegid.2009.01.008 19460312

[B42] Drummond AJ Rambaut A Shapiro B Pybus OG Bayesian coalescent inference of past population dynamics from molecular sequences Mol Biol Evol 2005 22 1185 1192 10.1093/molbev/msi103 15703244

[B43] Suchard MA Weiss RE Sinsheimer JS Bayesian selection of continuous-time Markov chain evolutionary models Mol Biol Evol 2001 18 1001 1013 11371589 10.1093/oxfordjournals.molbev.a003872

[B44] Drummond AJ Ho SY Phillips MJ Rambaut A Relaxed phylogenetics and dating with confidence PLoS Biol 2006 4 e88 10.1371/journal.pbio.0040088 16683862 PMC1395354

[B45] Worobey M Gemmel M Teuwen DE Haselkorn T Kunstman K Bunce M Muyembe JJ Kabongo JM Kalengayi RM Van Marck E Gilbert MT Wolinsky SM Direct evidence of extensive diversity of HIV-1 in Kinshasa by 1960 Nature 2008 455 661 664 10.1038/nature07390 18833279 PMC3682493

[B46] Guimaraes ML Vicente AC Otsuki K da Silva RF Francisco M da Silva FG Serrano D Morgado MG Bello G Close phylogenetic relationship between Angolan and Romanian HIV-1 subtype F1 isolates Retrovirology 2009 6 39 10.1186/1742-4690-6-39 19386115 PMC2680801

[B47] Bello G Guimaraes ML Passaes CP Almeida SE Veloso VG Morgado MG Short Communication: Evidences of Recent Decline in the Expansion Rate of the HIV Type 1 Subtype C and CRF31_BC Epidemics in Southern Brazil AIDS Res Hum Retroviruses 2009 25 1065 1069 10.1089/aid.2009.0106 19895209

[B48] Gomez Carrillo M Avila M Hierholzer J Pando M Martinez PL McCutchan FE Carr JK Mother-to-child HIV type 1 transmission in Argentina: BF recombinants have predominated in infected children since the mid-1980s AIDS Res Hum Retroviruses 2002 18 477 483 10.1089/088922202317406619 12015900

[B49] Robbins KE Lemey P Pybus OG Jaffe HW Youngpairoj AS Brown TM Salemi M Vandamme AM Kalish ML U.S. Human immunodeficiency virus type 1 epidemic: date of origin, population history, and characterization of early strains J Virol 2003 77 6359 6366 10.1128/JVI.77.11.6359-6366.2003 12743293 PMC155028

[B50] Walker PR Pybus OG Rambaut A Holmes EC Comparative population dynamics of HIV-1 subtypes B and C: subtype-specific differences in patterns of epidemic growth Infect Genet Evol 2005 5 199 208 10.1016/j.meegid.2004.06.011 15737910

[B51] Paraskevis D Magiorkinis E Magiorkinis G Sypsa V Paparizos V Lazanas M Gargalianos P Antoniadou A Panos G Chrysos G Sambatakou H Karafoulidou A Skoutelis A Kordossis T Koratzanis G Theodoridou M Daikos GL Nikolopoulos G Pybus OG Hatzakis A Multicentre Study on HIV Heterogeneity Increasing prevalence of HIV-1 subtype A in Greece: estimating epidemic history and origin J Infect Dis 2007 196 1167 1176 10.1086/521677 17955435

[B52] Blower S Behaviour change and stabilization of seroprevalence levels in communities of injecting drug users: correlation or causation? J Acquir Immune Defic Syndr 1991 4 920 923 1895214

